# Ursachen visueller Halluzinationen bei der Parkinson-Krankheit

**DOI:** 10.1007/s00115-021-01165-2

**Published:** 2021-08-03

**Authors:** Nico J. Diederich

**Affiliations:** Abteilung für Neurologie, Centre Hospitalier de Luxembourg, 4, rue Barblé, 1210 Luxemburg-Stadt, Luxemburg

**Keywords:** Parkinson-Krankheit, Visuelle Halluzinationen, Charles-Bonnet-Syndrom, Lhermitte-Syndrom, Copingstrategien, Parkinson’s disease, Visual hallucinations, Charles Bonnet syndrome, Lhermitte syndrome, Coping strategies

## Abstract

**Hintergrund:**

Visuelle Halluzinationen (VH) werden zumeist als Spätsymptome der Parkinson-Krankheit (PK) angesehen. Sie kommen jedoch in leichterer Form auch in Frühstadien der Erkrankung vor. Ursächlich wurden VH anfänglich als Folge einer dopaminergen Überstimulation gesehen, später ebenso im Rahmen einer demenziellen Entwicklung der PK.

**Fragestellung:**

Die vorliegende Arbeit untersucht, ob sich das Entstehungsmodell der VH in den letzten Jahren erweitert hat.

**Material und Methodik:**

Basierend auf klinischen, pharmakologischen und neuropathologischen Arbeiten sowie funktioneller Magnetresonanzgraphie erfolgt eine systematische Aufgliederung in monomodale und multimodale Entstehungsmodelle der VH. Die Anwendbarkeit auf unterschiedliche VH-Formen und -Auslösungsmomente wird jeweils kritisch beleuchtet.

**Ergebnisse:**

Einbußen bei der visuellen Informationsaufnahme und -verarbeitung, Defizite der Aufmerksamkeit und fehlerhafte Konnektivität zwischen kortikalen Netzwerken werden herausgearbeitet. Es bestehen z. T. Überlappungen mit dem Lhermitte-Syndrom und dem Charles-Bonnet-Syndrom. Kein Modell erklärt jedoch befriedigend alle Spielarten der VH. Nicht alle VH weisen die gleiche Pathogenese und stets eine schlechte Prognose auf.

**Schlussfolgerung:**

Die Ursachenkette visueller Halluzinationen ist komplex und individuell unterschiedlich. Inwieweit dies therapeutisch einsetzbar ist, ist bisher wenig erforscht. Es gibt erste Hinweise, dass neben einer Änderung der Medikation auch Visusverbesserung, die Einbindung des Partners/der Partnerin und vielleicht individuell anpassbare Copingstrategien erfolgreich eingesetzt werden könnten.

## Abstract (en)

**Background:**

Visual hallucinations (VH) have mainly been considered as late symptoms of Parkinson’s disease (PD); however, minor forms of VH also occur in early stages of the disease. Initially dopaminergic overstimulation was discussed as the cause and later on VH have been considered as an early red flag of dementia in PD.

**Objective:**

The present study analyzed whether the pathophysiological concept of VH has been enlarged in recent years.

**Material and methods:**

Clinical, pharmacological, neuropathological as well as functional magnetic resonance imaging studies dealing with VH were reviewed. A systematic classification in monomodal and multimodal models of VH is proposed. The applicability to various forms of VH and various triggering situations is critically examined.

**Results:**

Reduction of the visual information input, erroneous visual processing, attention deficits, and dysfunctional connectivity between various cerebral networks have been shown. There is partial overlapping with the Lhermitte syndrome and the Charles Bonnet syndrome. No model is able to fully explain all VH variants. Not all VH have the same pathogenesis and the same poor prognosis.

**Conclusion:**

The chain of causes underlying VH is complex and can vary from patient to patient. So far the therapeutic applications are largely unexplored; however, there is preliminary evidence that beside adjustment of the medication, improvement of visual acuity, active involvement of the partner, and possibly, individually adaptable coping strategies could be successfully implemented.

Visuelle Halluzinationen sind im späten Verlauf der Parkinson-Krankheit häufig und stellen eine Herausforderung für das Umfeld und den behandelnden Neurologen dar. Die zugrunde liegende Ursachenkette ist komplex und von Patient zu Patient unterschiedlich. Nicht nur die dopaminerge Medikation, sondern auch Aufmerksamkeitsstörungen, Vigilanzschwankungen und visuelle Defizite stellen Risikofaktoren dar. Bemerkenswerterweise setzen Patienten oft bereits ohne Fremdanleitung erfolgreich Copingstrategien ein.

Visuelle Halluzinationen (VH) gehören zu den häufigsten Spätkomplikationen der Parkinson-Krankheit (PK). Ihnen haftet der Ruf einer schlechten Prognose an, verbunden mit Einweisung ins Pflegeheim und höherer Mortalität [22]. Seit der Publikation einer Übersicht zu diesem Thema in *Der Nervenarzt* im Jahr 2003 [45] haben sich unsere Kenntnisse beträchtlich erweitert. Nicht nur kennen wir inzwischen weitere Spielarten der VH, sondern wir wissen auch, dass einige VH-Formen frühzeitig im Krankheitsverlauf auftreten können. Auch ist erkannt worden, dass die Ursachenkaskade vielschichtiger und komplexer ist als ursprünglich angenommen. Außer der üblicherweise erwogenen Levodopa-Psychose [47] werden nämlich Visuseinbußen, ungünstige Lichtverhältnisse, Störungen des Schlaf-wach-Rhythmus [14] und neuerdings zunehmend subtile Veränderungen innerhalb kognitiver Netzwerke sowie deren gestörter Informationsaustausch untereinander [50, 58, 62] ursächlich diskutiert. Kompensatorische Mechanismen können zur Anwendung kommen. Differenziertere Behandlungsstrategien, zugeschnitten auf das halluzinatorische Erleben des einzelnen Patienten, sind greifbarer geworden. Neben der üblichen Pharmakotherapie können auch nicht pharmakologische Strategien zur Anwendung kommen, wenngleich die Evidenzlage hier noch schwach ist. Im Gegensatz zu der früher stets als düster angesehenen Prognose der VH kann diese heute differenzierter und oft auch günstiger gestellt werden. Die vorliegende Arbeit vermittelt hierzu einen Überblick und gibt Ratschläge zur Umsetzung im Praxisalltag.

## Phänomenologie

Fénelon und Mitarbeiter haben vor 20 Jahren erstmals auf leichtere („Minor“-)Formen der Halluzination, nämlich „Vorbeihuschen“ und „Anwesenheit“, hingewiesen [16]. Das halluzinatorische Erleben einer „Anwesenheit“ ist sensu stricto keine VH, da der Patient angibt, eine fremde Person stehe hinter ihm; er nimmt diese also außerhalb seines Gesichtsfeldes oder „extrakampin“ wahr. Die Halluzination des „Vorbeihuschens“ wird vom Patienten zumeist als belanglos eingestuft. Er gibt sie oft auch erst auf wiederholtes Nachfragen an. Es kann sich um das flüchtige Vorbeigehen eines Menschen, das Vorbeifliegen eines Insekts oder die Wahrnehmung eines nicht identifizierbaren Schattens handeln [38]. Bemerkenswerterweise kommen solche leichteren Halluzinationen bereits bei unbehandelten „De-novo“-Patienten vor [51]. Die Illusion, also das Verkennen eines real wahrgenommenen Gegenstandes – z. B. wird ein Strauch als Mensch erfasst –, ist häufig an ungünstige Licht- oder Visusverhältnisse gebunden. Im Grenzbereich zwischen Illusion und Halluzination findet sich die selektive Diplopie als zumeist sehr kurzzeitige, doppelte Wahrnehmung isolierter Objekte oder Personen [48, 61]. Die klassischen VH sind ausgestaltet („formed“) und wiederholen sich nicht selten. Ihr Inhalt sind zumeist Tier- oder Menschengruppen; die emotionale Betroffenheit bleibt gering, ebenso der Ichbezug [2, 11]. Die meisten Fragebögen, so z. B. die Skala der MDS-UPDRS-Teil-Skala, unterscheiden, ob die Einsicht gewahrt bleibt (Pseudohalluzinationen nach alter Terminologie) oder nicht. Manche VH werden vom Patienten als „interessant“ erlebt, da sie den oft monotonen Tagesablauf bereichern [56]. Paranoides Verarbeiten der VH kommt eher selten vor. Die Themen Verfolgung, Bedrohung, Verarmung werden dann am häufigsten angegeben. Eine besonders belastende Spielart stellt das Capgras-Syndrom dar. Hierbei verliert der Patient die Anmutung der Vertrautheit einer bekannten Person, z. B. des Partners oder der Partnerin, wenngleich er dessen oder deren Physiognomie richtig erkennt. Infolgedessen ist er der Überzeugung, die gesehene Person sei ein Doppelgänger, der befremdlicherweise genau über ihn Bescheid wisse. Solche Verkennungssyndrome kommen aber auch bei anderen neurodegenerativen Prozessen vor, wenngleich häufiger bei Lewy-Körperchen-Erkrankungen [32]. Multimodale Halluzinationen, also visuell und auditorisch oder visuell und zönästhetisch, sind eher selten und werden hier nicht gesondert betrachtet [40]. Auch der Dermatozoenwahn mit ungünstiger Prognose soll hier nur am Rande angesprochen werden. Erwähnenswert ist hingegen, dass PK-Patienten und solche mit Lewy-Körperchen-Demenz das gleiche phänomenologische Spektrum der VH aufweisen [15, 46]. Wahneinfälle schließlich können eigenständig, also auch ohne VH, auftreten. Verfolgt und Bestohlen werden bilden übliche Themen. Das Othello-Syndrom kennzeichnet eine wahnhafte Eifersucht, und dem Partner bzw. der Partnerin wird dabei eine sexuelle Beziehung mit anderen unterstellt. Ein häufigeres Vorkommen bei eher jüngeren Männern, insbesondere unter Therapie mit Dopaminagonisten, wurde in diesem Zusammenhang beschrieben ([35]; Tab. 1).

**Table 1:** 

Symptom	Beschreibung	Ursache und Vorkommen
Gefühl der Anwesenheit („sensation de présence“) Extrakampine Halluzination	Eindruck eine andere, ungefährliche Person stehe hinter dem Patienten	Ungeklärt Zusammenhang mit REM-Schlafstörung diskutiert Bereits bei De-novo-PK-Patienten beschrieben *Ist eher selten*
Flüchtiges Vorbeihuschen („sensation de passage“)	Flüchtiger Eindruck, eine Person oder ein Schatten befinde sich im peripheren Gesichtsfeld oder ein Vogel/Insekt fliege vorbei; Patient erkennt zumeist sofort die Unwirklichkeit, berichtet darüber erst auf Anfrage	Ungeklärt Fehlinformation/Fehlinterpretation des visuellen Inputs Vorkommen ausschließlich im peripheren Gesichtsfeld Bereits bei De-novo-PK-Patienten beschrieben *Ist häufig, muss aber erfragt werden*
Visuelle Illusion	Tatsächlich wahrgenommenes Objekt wird fehlinterpretiert	Fehldeutung der Sinnhaftigkeit („salience“) Mangelhafte Lichtverhältnisse („Dämmerung“) oder emotionale Faktoren (Angst usw.) können das Auftreten begünstigen *Ist häufig*
Sonderform der visuellen Illusion: Pareidolie	Fehldeutung eines leblosen Objektes als bedrohliches Lebewesen	Möglicher Hinweis auf Lewy-Körperchen-Demenz *Ist eher selten*
Optische Halluzinationen im frontalen Gesichtsfeld	Menschen- oder Tiergruppe, oft repetitiv, oft nur flashartig auftretend, ohne Ichbezug	Unterschiedliche Ursachen, z. B. dopaminerge D_3_- und D_4_-Überstimulation; anticholinerge Blockierung; fehlerhafter visueller Input; Aufmerksamkeitsstörung *Ist häufig*
Selektive Diplopie	Einzelobjekt oder Einzelperson wird doppelt wahrgenommen	Unterschiedliche Mechanismen: z. B. Heterophorie; mangelhafte Fusion der retinal generierten Bilder; Aufmerksamkeitsstörung *Ist eher selten*
Akustische Halluzinationen	Selten isoliert; zumeist assoziiert mit visuellen Halluzinationen	Idem Fördernder Einfluss durch Presbyakusis möglich *Ist eher selten*
Taktile Halluzinationen („Dermatozoenwahn“)	Perzeption eines unangenehmen Hautkontakts, z. B. durch Insekten usw.	Oft wahnhafte Deutung Vorkommen bei demenzieller Entwicklung *Ist sehr selten*
Capgras-Syndrom	Bekannte Person (bes. Partner) wird als Doppelgänger betrachtet	Physiognomie wird richtig erkannt, Gefühl der Familiarität ist jedoch verloren Störung zwischen striatalem visuellem Kortex und limbischem System *Ist selten*
Wahneinfälle	Beziehen sich oft auf VH, können aber auch isoliert auftreten Verfolgung und Diebstahl sind Hauptthemen	Ungeklärt Oft Vorzeichen der demenziellen Entwicklung *Ist eher selten*
Sonderform der Wahneinfälle: Othello-Syndrom	Wahnhafte Eifersucht	Besonders bei jüngeren Männern Mögliche Nebenwirkung der Dopaminagonisten? *Ist sehr selten*

## Häufigkeit

Nicht alle Patienten berichten spontan über ihre VH, vielfach muss mehrfach nachgefragt werden [24]. Dies gilt nicht nur für die oben beschriebenen klassischen VH, sondern insbesondere auch für die Halluzinationen der „Anwesenheit“ und des „Vorbeihuschens“. Frühe Querschnittsstudien haben eine Häufigkeit von 15–38 % für komplexe VH ermittelt [1]. In Bezug auf die leichteren Halluzinationen wird sogar von einer Häufigkeit von 42 % bei De-novo-Patienten und von 50 % bei behandelten Patienten berichtet [51, 64]. Hier ist allerdings Vorsicht geboten, da diese Zahlen noch nicht bei größeren Kohorten bestätigt worden sind. Bei einer groß angelegten Langzeitstudie über 12 Jahre betrug die Häufigkeit der VH sogar 60 % [20]. Es ist möglich, dass in einem fortgeschrittenen Stadium der PK mit ausgeprägter Demenz die „kreative“ halluzinatorische Produktion wieder verblasst, in etwa vergleichbar der zunehmenden Anosognosie für mnestische Defizite bei fortgeschrittener Demenz. Allerdings ist dies bisher noch nicht statistisch belegt.

## Pathologie

Es waren als erstes Arbeiten zur Neuropathologie, die auf unterschiedliche Entstehungsmöglichkeiten der VH hinwiesen [27, 33]. Bei früh im Verlauf auftretenden VH finden sich Lewy-Körper (LK) in den Amygdala, insbesondere im basolateralen Kern und im Parahippokampus; bei späterer VH-Manifestation finden sich hingegen LK im Claustrum und Okzipitalkortex, in Verbindung mit einer Atrophie dieser Areale. Eine Hirnatrophie konnte auch in anderen Regionen nachgewiesen werden, u. a. in der oberen und lateralen Parietalregion und im lateralen Frontalkortex [18, 54]. Kasuistisch wurde bei Patienten mit Lewy-Körper-Demenz (LKD) auch über einen LK-Nachweis in der Retina berichtet [43]. Es ist bemerkenswert, dass bei Synukleinopathien mit VH autoptisch auch Amyloidablagerungen und neurofibrilläre Tangles und geminderte Konzentrationen von Amyloid Aβ-1–42 im Liquor nachweisbar sind [30]. Bislang fehlen allerdings systematische Studien zum Krankheitsbefall von Hirnregionen, die für Vigilanz, Aufmerksamkeit und visuelle Verarbeitung verantwortlich sind. Da zudem die meisten Autopsien bei Patienten im Spätstadium durchgeführt werden, könnten Atrophie und LK-Befall auch durch allgemeines Fortschreiten der Erkrankung und Alterungsprozesse erklärbar sein. Deshalb sind die Daten von in vivo durchführbaren Bildgebungsverfahren ggf. sogar während des Erlebens einer VH als spezifischer anzusehen. Solche Befunde werden weiter unten angeführt.

## Monomodale Modelle

Der klinische Alltag suggeriert vordergründig monomodale Erklärungsmodelle der VH. Handelt es sich nicht einfach um eine dopaminerge Psychose, früher auch Levodopapsychose genannt, da die Reduktion dieser Medikation oft Erleichterung bringt? Da VH gehäuft nachts auftreten, wurde spekuliert, ob eine Verbindung mit gestörtem REM-Schlaf bestehen könnte [3]. Auch wurde postuliert, dass eine Dysfunktion des visuellen Systems eine entscheidende Rolle spielen könnte. Allerdings decken bei näherer Analyse diese „simplen“ Erklärungsmodelle nicht das gesamte Spektrum der VH ab. Daher sind eine komplexere Ursachenverflechtung, vielleicht aber auch unterschiedliche Entstehungsweisen für unterschiedliche Arten der VH zu erwägen. Die wichtigsten monomodalen ätiologischen Hypothesen werden trotzdem kritisch im Folgenden dargestellt, um später auch die multimodalen Modelle der Entstehung besser verstehen zu können.

### Die dopaminerge Psychose

Die Arbeitsgruppe um Harald Klawans und Christopher Goetz postulierte in den 1970er-Jahren, dass die kontinuierliche dopaminerge Medikation zur Überstimulation mesolimbischer D3- und D4-Rezeptoren führt [47]. Die Auslösung eines „Kindling“-Phänomens wäre die Folge: Dopaminrezeptoren würden nach längerer Therapiedauer überempfindlicher, sodass selbst geringe Dopamindosen VH auslösen könnten. Auch wurde ein Ungleichgewicht verschiedener Neurotransmitter diskutiert. Insbesondere bei der LKD weisen quantitative neurochemische Autopsiebefunde auf einen Verlust cholinerger Neurone im Neokortex hin. Geht deren Filterfunktion bezüglich nicht relevanter interner oder externer sensorischer Informationen verloren, so werden diese Informationen nicht mehr als „Hintergrundrauschen“ verarbeitet, sondern als VH in die bewusste Wahrnehmung aufgenommen [52]. Ebenso könnte die Levodopatherapie einen relativen Serotoninmangel verursachen und sekundär eine postsynaptische Serotoninrezeptorüberempfindlichkeit hervorrufen. Neurotoxikologische Befunde nach LSD-Einnahme legen hier eine besondere Rolle des 5‑HT2A-Rezeptors nahe [41]. So wurde bei PK-Patienten mit VH über eine reduzierte postsynaptische Darstellung dieses Rezeptors berichtet [4].

Im klinischen Alltag hat sich die Anwendbarkeit dieser Transmitterhypothesen bewährt. Die Reduktion von Medikamenten mit anticholinerger Wirkung, die Reduktion der dopaminergen Medikation, besonders der DA-Agonisten mit D3- und D4-Angriffspunkt, oder auch der Einsatz von Clozapin und Pimavanserin, die u. a. als Antagonisten des 5‑HT2A-Rezeptors agieren, können die VH reduzieren. Der Einsatz der atypischen Neuroleptika wurde im Detail in *Der Nervenarzt* beschrieben [28].

Jedoch gibt es auch zahlreiche Argumente, die diese „Monopolstellung“ der pharmakologischen Erklärungsmodelle infrage stellen. So wurde bereits *vor* der Levodopaära über VH bei PK-Patienten berichtet [17]. Die Halluzinationen des „Vorbeihuschens“ und der „Anwesenheit“ sind auch bei unbehandelten De-novo-PK-Patienten nachweisbar [51]. Ebenso berichten LKD-Patienten über VH auch ohne dopaminerge Medikation [15, 46]. Nicht zuletzt scheiterte ein VH-Triggerversuch: PK-Patienten, die zu Hause häufig VH erlebten, berichteten erstaunlicherweise nicht über VH bei intravenöser Applikation einer hohen Levodopadosis im Labor [23].

### VH als Traumintrusion in den Wachzustand

Im Jahr 1922 berichtete Jean Lhermitte [39], dass Patienten mit pedunkulären Hirnstammläsionen insbesondere vaskulärer Natur über geformte, farbige und sehr lebhafte VH klagten, die sie häufig auch wahnhaft verarbeiteten. Diese Patienten wiesen deutliche Störungen des Schlaf-wach-Rhythmus auf. Lhermitte charakterisierte den prototypischen Patienten mit pedunkulären Halluzinationen als einen „wachen oder nur unvollständig schlafenden Träumer“. Erst viel später wurde die Schlüsselfunktion des dorsolateralen Nucleus geniculatus erkannt [41]. Üblicherweise unterliegt diese Struktur dem hemmenden Einfluss der Raphé-Kerne und des Nucleus pedunculopontinus (NPP). Beim Fehlen dieser Hemmung kommt es zur ungebremsten Erregung verschiedener Thalamuskerne und des Kortex. Im Falle der PK könnte ein solcher Mechanismus tatsächlich aktiv werden, da bereits in vivo eine Atrophie des NPP nachweisbar ist und die Autopsie einen zusätzlichen LK-Befall dieses Kerns anzeigt [31, 57]. Einschränkend ist jedoch festzustellen, dass PK-Patienten zumeist keine Unterschiede zwischen Tag- und Nachtzeit in Bezug auf Erscheinungsformen der VH aufweisen. Mittels Polysomnographie konnte aber ein Auftreten der VH unmittelbar im Anschluss an eine REM-Schlaf-Phase beobachtet werden [3]. Auch legen Korrelationsstudien einen Zusammenhang zwischen VH und REM-Schlaf-Verhaltensstörung nahe [1]. Es bestehen also Gemeinsamkeiten von einerseits dem Lhermitte-Syndrom und andererseits den VH bei der PK. So ist in beiden Fällen eine Intrusion von REM-Schlaf-Fragmenten in den Wachzustand zu postulieren; die Neuropathologie deutet in beiden Fällen auf Läsionen der „Traumfabrik“ und der Schlafregulierungszentren hin [49]. Doch auch diese Hypothese kann nicht sämtliche VH erklären. So weisen z. B. die VH des PK-Patienten nicht die bizarren Charakteristika des REM-Schlafs auf. Zudem erleben PK-Patienten VH auch bei vollem Bewusstsein.

### VH infolge visueller sensorischer Deprivation

Charles Bonnet beschrieb VH bei seinem unter einer Katarakt leidenden, aber geistig noch regen Großvater. Der ältere Herr hatte komplexe, stereotype, sich oft wiederholende VH, die plötzlich auftraten und ebenso rasch wieder verschwanden. Seitdem beschreibt das Charles-Bonnet-Syndrom solche flashartig auftretenden VH bei älteren Patienten mit zumeist reduzierter Sehkraft und gelegentlich auch eingeschränkten kognitiven Funktionen [59]. PK-Patienten weisen unterschiedliche Einbußen des Sehens hinsichtlich Kontrasterkennen und Farbdiskrimination auf [2, 53]. Ein Zusammenhang mit dem Auftreten von VH konnte sowohl bei nicht dementen PK-Patienten als auch bei LKD-Patienten nachgewiesen werden [10, 42]. Die Verarbeitung des visuellen Inputs ist ebenso gestört. PK-Patienten mit VH benötigen eine längere Reaktionszeit als PK-Patienten ohne VH, um einen visuellen Input bewusst wahrzunehmen [37]. Die aufsteigende („bottom-up“) visuelle Verarbeitung ist gestört. Mit funktioneller Magnetresonanztomographie wurde nachgewiesen, dass bei PK-Patienten mit VH vor dem bewussten Bilderkennen Areale des Okzipitalkortex, des Gyrus fusiformis und der Frontalregion weniger ausgeprägt und verzögert aktiviert werden, als dies bei PK-Patienten ohne VH der Fall ist [44]. Die verminderte Aktivierung des Gyrus fusiformis und des Gyrus lingualis hält auch während des Bilderkennens an. Diese Befunde deuten auf eine ursächlich wirkende partielle visuelle Deprivation hin, ähnlich wie dies beim Charles-Bonnet-Syndrom der Fall ist [36]. Der qualitativ mangelhafte visuelle Input ermöglicht das Auftauchen („popping up“) gespeicherter oder sui generis generierter Bilder in das Bewusstsein. Strenge Gütekriterien bezüglich der perzeptuellen Wirklichkeit eines Inputs sind damit aufgegeben [7].

In den letzten Jahren häuften sich die Befunde in Bezug auf den mangelhaften visuellen Input bei der PK [26, 63]. PK-Patienten mit VH weisen eine dünnere retinale Nervenfaserschicht auf als PK-Patienten ohne VH [36], möglicherweise sekundär nach Befall der retinalen ganglionären Zellschicht [34]. Beim Vorliegen von VH zeigt die Magnetspektroskopie eine reduzierte GABA-Konzentration im primären visuellen Kortex. Da reduzierte GABA-Spiegel die visuelle Verarbeitung eines bruchstückhaften Inputs „glätten“, kommt es häufiger zur Fehlinterpretation von mehrdeutigen Inputs [19].

Das Konzept des Charles-Bonnet-Syndroms erklärt nur teilweise VH bei der PK. Zwar ist in beiden Szenarien der primäre visuelle Input mangelhaft und die emotionale Betroffenheit gering. Auch kann die Einsicht gewahrt bleiben. Im experimentellen Setting jedoch vermag Flackerlicht bei PK-Patienten mit intakter Aufmerksamkeit lediglich nichtfigürliche VH hervorrufen, nicht aber VH von lebenden Gestalten [65]. Das Modell liefert auch keine Erklärung für sekundäre paranoide Deutungen.

## Multimodale Modelle (Abb. 1, 2 und 3)

**Figure 1:**
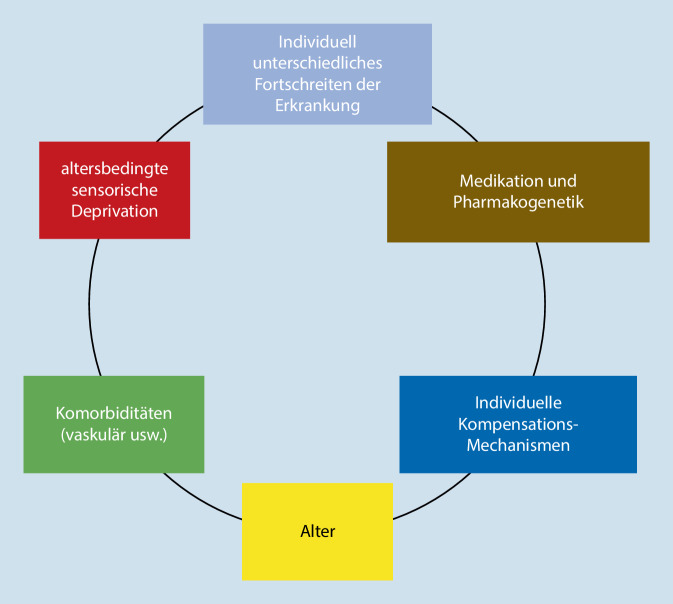

Wie gezeigt, sind die monomodalen Erklärungsversuche zwar attraktiv, da einfach zu belegen und oft klinisch auch sofort anwendbar. Jedoch vermag kein Modell die individuell unterschiedliche Suszeptibilität für VH bei PK-Patienten, den andersartigen Verlauf – benigne oder sich zur Psychose entwickelnd – oder die Variabilität der Triggerfaktoren zu erklären. Das Zusammenspiel mehrerer Risikofaktoren ist zwar einfach zu modellieren (Abb. 1), kann aber auch nicht im Einzelfall das Vorhandensein von VH erklären. Es wurden deshalb komplexere Modelle entwickelt. Diese weisen nach, dass bei perzeptiven Defiziten, welcher Ursache auch immer, durch „Top-down“-Mechanismen gegengesteuert werden kann. Interaktionen auf unterschiedlichen Ebenen sind möglich. Diesen Einflussmöglichkeiten wird in den nun zu besprechenden multifaktoriellen Modellen Rechnung getragen. Sie sollen sowohl dem Forscher als auch dem Kliniker helfen, weitere Gesichtspunkte der VH bei PK-und LKD-Patienten nicht nur zu erkennen, sondern diese als Therapieansätze zu nutzen. Folgende Modelle werden erläutert:

- das Aktivierungs-Input-Modulations-Modell (AIM) von Hobson,
- das Modell der Perzeptions- und Aufmerksamkeitsdefizite,
- das Modell zur Aufmerksamkeitskontrolle,
- die Hypothese der thalamokortikalen Dysregulation (TKD).

**Figure 2:**
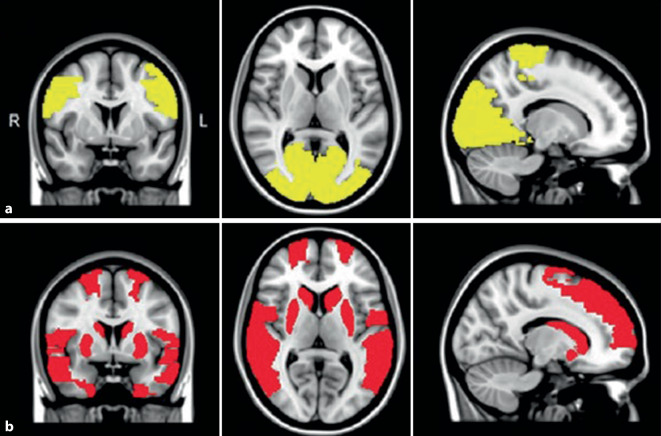

**Figure 3:**
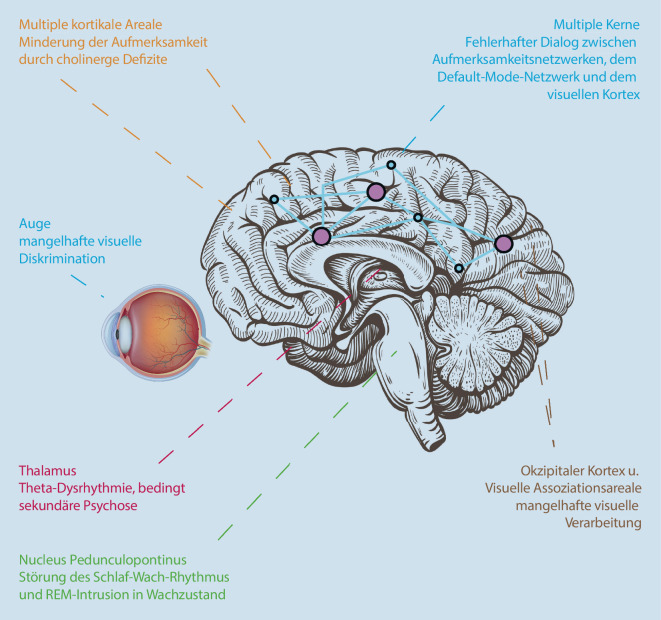

Die Modelle werden in der Chronologie ihrer Veröffentlichung vorgestellt.

### Das AIM-Modell nach Hobson

Dies ist ein dreidimensionales Modell [13]. Die Aktivierung stellt die erste Achse dar; sie reicht vom tiefen NREM-Schlaf über REM-Schlaf bis zum Wachzustand. Der Input beschreibt den Informationsaustausch mit der Außenwelt. Letzterer schwankt abhängig davon, ob die „Schleusen zur Außenwelt geöffnet oder geschlossen“ sind. Das System lässt aber eine gewisse „Porosität“ zu. Die Modulation integriert über die Zeitachse aminerge (Noradrenalin und Serotonin) und cholinerge Einflüsse und beschreibt außerdem den möglichen Einfluss einer Medikation. Unregelmäßigkeiten können in diesem Modell sowohl die A‑Achse (reduzierte Vigilanz), die I‑Achse (geminderte Stärke des visuellen Inputs) oder ebenso die M‑Achse (Ungleichgewicht der Transmitter und Medikamenteneinfluss) betreffen. Störungen berühren auch das Filtern externer Bilder bzw. die Generierung interner Bilder [13]. Die multifaktorielle Genese der VH, welche das Modell vorschlägt, konnte in einer Kohorte von 84 PK-Patienten, davon ein Drittel mit VH und zwei Drittel ohne VH, bestätigt werden [21]. So wurden als VH-bestimmende Faktoren multimodal u. a. die visuelle Perzeptionsstärke, kognitive Einbußen sowie eine REM-Schlaf-Verhaltensstörung erkannt. Die praktische Anwendbarkeit des AIM-Modells ist also gegeben. Geminderte Inputstärke z. B. bei Dämmerlicht bewirkt illusionäre Verkennungen eines Gegenstandes; Intensitätszunahme der intern generierten Bilder führt zur REM-Intrusion in den Wachzustand; mangelhafter visueller Input mit gleichzeitiger Akzeptanz intern gebildeter Bilder produziert VH in normaler Umgebung [13]. Auch für die selektive Diplopie werden sich überlappende Mechanismen diskutiert, so z. B. Heterophorie bei Ermüdung als auch reduzierte Aufmerksamkeit oder mangelhafte Fusion der beidseits retinal generierten Bilder [48, 61]. Das Modell kann somit zwar VH unter sehr unterschiedlichen Bedingungen erklären; es vermag jedoch weder deren sich wiederholenden Inhalt noch die Entwicklung einer paranoiden Psychose zu erklären.

### Das Modell der Perzeptions- und Aufmerksamkeitsdefizite

In Bezug auf die visuelle Wahrnehmung trennt dieses Modell klar die Ebenen „bottom up“ und „top down“ [9]. Liefert erstere Ebene einen qualitativ mangelhaften visuellen Input, so treten sog. „Proto-Objekte“ unbewusst als Ersatz in Erscheinung. Sie werden teils neu generiert, teils aus dem Gedächtnisspeicher abgerufen. Bei geminderter Aufmerksamkeit werden sie unkritisch als reale Objekte akzeptiert, sprich als VH wahrgenommen. Dieses Modell stellt geminderte Aufmerksamkeit als entscheidenden Faktor bei der VH-Entstehung in den Vordergrund. Die Minderung kann verursacht werden durch kortikale cholinerge Defizite [52] oder auch durch die Einnahme von Anticholinergika. Das Modell vermag aber weder den VH-Inhalt zu erklären noch die klinische Entwicklung der VH vorherzusagen.

### Das Modell zur Aufmerksamkeitskontrolle

Bedingt durch die rasante Entwicklung der funktionellen Magnetresonanztomographie (fMRT) ist das Modell zur Aufmerksamkeitskontrolle am besten experimentell belegt [58]. Es legt zuerst dar, dass normalerweise das ventrale Aufmerksamkeitsnetzwerk („ventral attention network“) die Bedeutung oder Sinnhaftigkeit („salience“) eines vorerst noch nicht gedeuteten Inputs überprüft. Hierzu ist ein „Dialog“ mit dem im Ruhezustand überwiegenden „Default-mode“-Netzwerk erforderlich. Erst dann erkennt schließlich das dorsale Aufmerksamkeitsnetzwerk korrekt den visuellen Input. Bei PK-Patienten mit VH funktionieren diese „Zwiegespräche“ zwischen den einzelnen Netzwerken nicht mehr und es kommt zu Fehlbestimmungen, so z. B. zur illusionären Verkennung eines Gegenstandes. Dieses Modell kann besonders die Entstehung von Illusionen gut erklären. Abnorm gesteigerte Konnektivität zwischen dem „Default-mode“-Netzwerk und dem visuellen Kortex könnte begründen, warum „Gedankenabschweifen“ („mind wandering“) bei PK-Patienten mit VH ausgeprägter ist als bei solchen ohne VH [62]. Eine fehlerhafte Konnektivität zwischen den Netzwerken wird auch bei der Entstehung der leichteren VH angenommen [6] und dürfte damit generell einen früh einsetzenden pathophysiologischen Mechanismus bei der PK darstellen. Letztlich ist es aber fast unmöglich, sämtliche beobachtete Minderungen der funktionellen Konnektivität (Abb. 2; [29]) in ein einziges Modell zu integrieren.

### Die thalamokortikale Dysrhythmie

Die jüngste Hypothese postuliert einen Zusammenhang zwischen der thalamokortikalen Dysrhythmie (TKD) und VH bei Patienten mit Parkinson-Demenz oder mit LKD [49]. Entsprechend dieser Annahme hemmt die in Thalamuskernen generierte Theta-Dysrhythmie frontale Aufmerksamkeitsnetzwerke und führt damit zur Entkopplung mit dem „Default-mode“-Netzwerk. Dessen Eigenaktivität wird nun nicht mehr kontrolliert und führt zu willkürlichen Verbindungen zwischen autobiografischen Erinnerungsfragmenten und dem noch zu identifizierenden visuellen Input. Dies wiederum bedingt illusionäre Verkennungen, z. B. von Gegenständen als bekannte Personen (Pareidolien), und schließlich wahnhafte Interpretationen des Erlebten. Die Autoren sehen auch einen Zusammenhang zwischen der TKD und ähnlichen Theta-Rhythmen im REM-Schlaf von PK-Patienten. Sie bestätigen damit indirekt die Hypothese der VH als REM-Intrusion in den Wachzustand. Den posterioren Thalamuskernen und dem posterioren zingulären Kortex kommt hierbei entscheidende pathogenetische Bedeutung zu, da diese Areale LK-beladen sind und/oder atrophisch geworden sind. Zudem unterliegen sie bei der PK der bereits oben erwähnten fehlerhaften Stimulation durch den NPP. Dieses Modell erscheint am besten geeignet, um das sekundär-paranoide Erleben zu erklären, ist aber ebenso wie die vorher aufgeführten Modelle ungeeignet, alle Spielarten der VH zu erklären.

Eine Synthese im Bild der wichtigsten VH-induzierenden Faktoren wird in Abb. 3 vorgeschlagen.

## Kompensatorische Mechanismen

Diese Übersicht geht nicht auf die Pharmakotherapie der VH ein, da diese kürzlich und erschöpfend in *Der Nervenarzt* erörtert worden sind [28]. Der Streifzug durch monomodale und multifaktorielle Erklärungsmodelle mag manchem Kliniker theoretisch und praxisfern vorgekommen sein. Bei näherer Betrachtung zeigt sich allerdings, dass diese Modelle uns helfen, zu verstehen, wie PK-Patienten mit halluzinatorischem Erleben umgehen (können).

Bereits früh wurde darauf hingewiesen, dass PK-Patienten problemorientierte und selbst entwickelte Copingstrategien (CS) erfolgreich nutzen [25]. Die Anwendung auf VH wurde in zwei Studien untersucht; hier wandten zwischen 53 und 78 % der PK-Patienten CS an, die sie eigenständig oder unter klinischer Anleitung erprobt hatten ([5, 12]; Tab. 2). CS können visueller, kognitiver oder interaktiver Art sein. So mag der Patient das halluzinierte Objekt nochmals aus nächster Nähe betrachten, um dann dessen Unwirklichkeit erkennen zu können. Er überprüft oder verbessert damit die visuelle Eingabe. Auch kann der nicht stattgefundene interne „Dialog“ zwischen den zerebralen Netzwerken durch ein zwischenmenschliches Gespräch ersetzt werden (Frage an den/die Partner/in: „Siehst du dies auch?“). Hier zeigt sich also eine direkte Anwendbarkeit der multimodalen Erklärungsmodelle. Absichtlich werden auch andere sensorische Modalitäten aufgerufen (z. B. mit dem Stock das vermeintliche Objekt ertasten, den visuell Halluzinierten ansprechen und dann beruhigt sein, wenn keine Antwort erfolgt; [12]). Leider gibt es hierzu bisher nur die zwei erwähnten Beobachtungsstudien, jedoch keine kontrollierte Studie „Intervention“ versus „keine Intervention“. Wenn auch oft in kleinen Gruppen durchgeführt, gibt es hingegen zahlreiche Untersuchungen zum Einsatz kognitiver Verhaltenstherapie („cognitive-behavioral treatment“) bei akustischen Halluzinationen der Schizophrenie [60].

**Table 2:** 

*Kognitive Strategien 53–69* *%* [5, 12]
– Rationalisierung des Erlebten durch Selbstreflektion
*Aktionsbasierte Strategien 65* *%* [12]
– Erforschen des Ortes der VH mithilfe anderer sensorischer Modalitäten (Ansprechen, mit einem Stock betasten usw.)
*Interaktive Strategien 41–62* *%* [5, 12]
– Diskussion des Erlebten mit Betreuer – Ansprechen der begleitenden Emotionen (z. B. Angst) mit Betreuer
*Visuelle Strategien 33* *%* [5]
– Augenreiben – Visusverbesserung (Brille; Lichtschalter) – Verdächtiges Objekt aus näherer Entfernung nochmals betrachten
*Humor 35* *%* [12]
– Humorvolle, distanzierte Schilderung des halluzinatorischen Erlebens

Es ist wahrscheinlich, dass aktive Kompensationsmechanismen – im Gegensatz zu einer Haltung des Negierens oder des „Für-sich-Behaltens“ – die Lebensqualität der PK-Patienten verbessern [8]. Gewinnt der Patient früh und möglichst dauerhaft Einsicht in seine gestörte Wahrnehmung sowie den Umgang damit und wird er hierbei von Familienangehörigen aktiv unterstützt, so ist der Leidensdruck und das Stigma geringer, als wenn dies nicht geschieht. Auch kann man dann ggf. den Einsatz atypischer Neuroleptika hinauszögern oder ganz vermeiden. All dies setzt die regelmäßige Einbindung und Aufklärung der Angehörigen voraus, nicht zuletzt um zudem deren Stressniveau zu senken [55].

Der Vollständigkeit halber soll erwähnt werden, dass PK-Patienten selten einen Krankheitsgewinn aus dem VH-Erleben ziehen können. Wie eingangs angeführt, sehen einige Patienten VH als freudige Abwechslung in einem ansonsten trübem Alltag an [56]. Es wurde aber bisher nicht untersucht, ob PK-Patienten durch Eigensuggestion sog. eidetische Trugwahrnehmungen erleben können. Allerdings sind die meisten PK-Patienten der Ansicht, dass sie das VH-Erleben wenig beeinflussen oder gar steuern können [5].

## Diskussion und Ausblick

Die Vielschichtigkeit möglicher Ursachen der VH bei der PK ist ausführlich dargestellt und erläutert worden. Es zeigt sich, dass die Erforschung der Ursachenkette sich zunehmend komplexen Netzwerkstörungen widmet. Einfachere Rezeptorhypothesen sind weitgehend verlassen worden. Auch wurde erörtert, dass es unterschiedliche Spielarten der VH gibt, mit wechselnder Ursachenverflechtung und variabler prognostischer Bedeutung. Vereinfachungen wie etwa: VH sind stets ein Signum mali ominis oder VH können nur medikamentös angegangen werden, klingen zwar im klinischen Alltag verlockend, können aber auch zu vorschnellen prognostischen Aussagen und zum mangelhaften Ausschöpfen aller therapeutischen Möglichkeiten führen.

Es erscheint vielversprechend, in Zukunft die VH-Genese in Bezug auf Fehlfunktionen des Visus und Störungen des Schlafs präziser zu erforschen. Es ist anzunehmen, dass damit auch Medikamente, die nicht auf das dopaminerge, sondern z. B. auf das cholinerge oder auf das serotoninerge System Einfluss nehmen, individueller oder „personalisiert“ beim PK-Patienten mit VH zum Einsatz kommen können. Nicht zuletzt sollten Copingstrategien methodisch sauber erforscht werden, um damit Einzug in das therapeutische Routinearsenal zu finden.

## Fazit für die Praxis

- Die Ursachenkette visueller Halluzinationen bei der PK ist komplexer als bisher angenommen.
- Dies erklärt z. B., warum nicht alle Patienten bei einer bestimmten Medikamentendosis visuelle Halluzinationen erleben.
- Besonders Visus- und Aufmerksamkeitsdefizite rücken ins Blickfeld.
- Funktionelle Magnetresonanztomographie kann fehlerhafte Netzwerkinteraktionen zeigen.
- Mit dem Patienten kann die Möglichkeit selbst anwendbarer Copingstrategien besprochen werden. Diese nichtmedikamentösen Behandlungsmethoden bedürfen aber noch weiterer wissenschaftlicher Validierung.

## References

[1] Aarsland D, Larsen JP, Cummings JL (1999). Prevalence and clinical correlates of psychotic symptoms in Parkinson disease: a community-based study. Arch Neurol.

[2] Archibald NK, Clarke MP, Mosimann UP (2011). Visual symptoms in Parkinson’s disease and Parkinson’s disease dementia. Mov Disord.

[3] Arnulf I, Bonnet AM, Damier P (2000). Hallucinations, REM sleep, and Parkinson’s disease: a medical hypothesis. Neurology.

[4] Ballanger B, Strafella AP, van Eimeren T (2010). Serotonin 2A receptors and visual hallucinations in Parkinson disease. Arch Neurol.

[5] Barnes J, Connelly V, Boubert L (2013). Behavioural coping patterns in Parkinson’s patients with visual hallucinations. J Neuropsychol.

[6] Bejr-Kasem H, Pagonabarraga J, Martínez-Horta S (2019). Disruption of the default mode network and its intrinsic functional connectivity underlies minor hallucinations in Parkinson’s disease. Mov Disord.

[7] Bowman AR, Bruce V, Colbourn CJ (2017). Compensatory shifts in visual perception are associated with hallucinations in Lewy body disorders. Cogn Res Princ Implic.

[8] Bucks RS, Cruise KE, Skinner TC (2011). Coping processes and health-related quality of life in Parkinson’s disease. Int J Geriatr Psychiatry.

[9] Collerton D, Perry E, McKeith I (2005). Why people see things that are not there: a novel perception and attention deficit model for recurrent complex visual hallucinations. Behav Brain Sci.

[10] Diederich NJ, Goetz CG, Raman R (1998). Poor visual discrimination and visual hallucinations in Parkinson’s disease. Clin Neuropharmacol.

[11] Diederich NJ, Pieri V, Goetz CG (2000). Die optischen Halluzinationen des Parkinson-Patienten und das Charles Bonnet-Syndrom. Fortschr Neurol Psychiatr.

[12] Diederich NJ, Pieri V, Goetz CG (2003). Coping strategies for visual hallucinations in Parkinson’s disease. Mov Disord.

[13] Diederich NJ, Goetz CG, Stebbins GT (2005). Repeated visual hallucinations in Parkinson’s disease as disturbed external/internal perceptions: focused review and a new integrative model. Mov Disord.

[14] Diederich NJ, Goetz CG, Stebbins GT, Collerton D, Mosimann UP, Perry E (2015). The pathology of hallucinations: one or several points of processing breakdown?. The neuroscience of visual hallucinations.

[15] Dodel R, Koschel J, Lorenzl S (2018). Demenz mit Lewy-Körpern. Fortschr Neurol Psychiatr.

[16] Fénelon G, Mahieux F, Huon R (2000). Hallucinations in Parkinson’s disease: prevalence, phenomenology and risk factors. Brain.

[17] Fénelon G, Goetz CG, Karenberg A (2006). Hallucinations in Parkinson disease in the prelevodopa era. Neurology.

[18] Ffytche D, Creese B, Politis M (2017). The psychosis spectrum in Parkinson disease. Nat Rev Neurol.

[19] Firbank MJ, Parikh J, Murphy N (2018). Reduced occipital GABA in Parkinson disease with visual hallucinations. Neurology.

[20] Forsaa EB, Larsen JP, Wentzel-Larsen T (2010). A 12-year population-based study of psychosis in Parkinson disease. Arch Neurol.

[21] Gallagher DA, Parkkinen L, O’Sullivan SS (2011). Testing an etiological model of visual hallucinations in Parkinson’s disease. Brain.

[22] Goetz CG, Stebbins GT (1995). Mortality and hallucinations in nursing home patients with advanced Parkinson’s disease. Neurology.

[23] Goetz CG, Pappert EJ, Blasucci LM (1998). Intravenous levodopa in hallucinating Parkinson’s disease patients: high-dose challenge does not precipitate hallucinations. Neurology.

[24] Haeske-Dewick HC (1995). Hallucinations in Parkinson’s disease: characteristics and associated clinical features. Int J Geriatr Psychiatry.

[25] Haltenhof H, Krakow K, Zöfel P (2000). Krankheitsverarbeitung bei Morbus Parkinson. Nervenarzt.

[26] Han G, Han J, Han K (2020). Visual acuity and development of Parkinson’s disease: a nationwide cohort study. Mov Disord.

[27] Harding AJ, Broe GA, Halliday GM (2002). Visual hallucinations in Lewy body disease relate to Lewy bodies in the temporal lobe. Brain.

[28] Haussmann R, Bauer M, Donix M (2016). Evidenz zur Behandlung der Parkinson-assoziierten Psychose. Nervenarzt.

[29] Hepp DH, Foncke EM, Olde Dubbelink KT (2017). Loss of functional connectivity in patients with Parkinson disease and visual hallucinations. Radiology.

[30] Jacobson SA, Morshed T, Dugger BN (2014). Plaques and tangles as well as Lewy-type alpha synucleinopathy are associated with formed visual hallucinations. Parkinsonism Relat Disord.

[31] Janzen J, van’t Ent D, Lemstra AW (2012). The pedunculopontine nucleus is related to visual hallucinations in Parkinson’s disease: preliminary results of a voxel-based morphometry study. J Neurol.

[32] Josephs KA (2007). Capgras syndrome and its relationship to neurodegenerative disease. Arch Neurol.

[33] Kalaitzakis ME, Christian LM, Moran LB (2009). Dementia and visual hallucinations associated with limbic pathology in Parkinson’s disease. Parkinsonism Relat Disord.

[34] Kassubek J, Danek A, Del Tredici-Braak K (2013). Das Auge als Zugang zur Pathophysiologie von Parkinson-Syndromen. Nervenarzt.

[35] Kataoka H, Sugie K (2018). Delusional jealousy (Othello syndrome) in 67 patients with parkinson’s disease. Front Neurol.

[36] Lee JY, Kim JM, Ahn J (2014). Retinal nerve fiber layer thickness and visual hallucinations in Parkinson’s disease. Mov Disord.

[37] Lefebvre S, Baille G, Jardri R (2016). Hallucinations and conscious access to visual inputs in Parkinson’s disease. Sci Rep.

[38] Lenka A, Pagonabarraga J, Pal P (2019). Minor hallucinations in Parkinson disease: a subtle symptom with major clinical implications. Neurology.

[39] Lhermitte J (1922). Syndrome de la calotte pédonculaire. Les troubles psychosensoriels dans les lésions du mésencéphale. Rev Neurol (Paris).

[40] Llorca PM, Pereira B, Jardri R (2016). Hallucinations in schizophrenia and Parkinson’s disease: an analysis of sensory modalities involved and the repercussion on patients. Sci Rep.

[41] Manford M, Andermann F (1998). Complex visual hallucinations: clinical and neurobiological insights. Brain.

[42] Matar E, Phillips JR, Martens KAE (2019). Impaired colour discrimination—A specific marker of hallucinations in Lewy body disorders. J Geriatr Psychiatry Neurol.

[43] Maurage CA, Ruchoux MM, de Vos R (2003). Retinal involvement in dementia with Lewy bodies: a clue to hallucinations?. Ann Neurol.

[44] Meppelink AM, de Jong BM, Renken R (2009). Impaired visual processing preceding image recognition in Parkinson’s disease patients with visual hallucinations. Brain.

[45] Moser A, Hagenah J, Kömpf D (2003). Halluzinationen beim Morbus Parkinson. Nervenarzt.

[46] Mosimann UP, Rowan EN, Partington CE (2006). Characteristics of visual hallucinations in Parkinson disease dementia and dementia with Lewy bodies. Am J Geriatr Psychiatry.

[47] Moskovitz C, Moses H, Klawans HL (1978). Levodopa-induced psychosis: a kindling phenomenon. Am J Psychiatry.

[48] Nebe A, Ebersbach G (2007). Selective diplopia in Parkinson’s disease: a special subtype of visual hallucination?. Mov Disord.

[49] Onofrj M, Bonanni L, Albani G (2006). Visual hallucinations in Parkinson’s disease: clues to separate origins. J Neurol Sci.

[50] Onofrj M, Espay AJ, Bonanni L (2019). Hallucinations, somatic-functional disorders of PD-DLB as expressions of thalamic dysfunction. Mov Disord.

[51] Pagonabarraga J, Martinez-Horta S, Fernández de Bobadilla R (2016). Minor hallucinations occur in drug-naive Parkinson’s disease patients, even from the premotor phase. Mov Disord.

[52] Perry EK, Perry RH (1995). Acetylcholine and hallucinations: disease-related compared to drug-induced alterations in human consciousness. Brain Cogn.

[53] Pieri V, Diederich NJ, Raman R (2000). Decreased colour discrimination and contrast sensitivity in Parkinson’s disease. J Neurol Sci.

[54] Ramírez-Ruiz B, Martí MJ, Tolosa E (2007). Cerebral atrophy in Parkinson’s disease patients with visual hallucinations. Eur J Neurol.

[55] Renouf S, Ffytche D, Pinto R (2018). Visual hallucinations in dementia and Parkinson’s disease: a qualitative exploration of patient and caregiver experiences. Int J Geriatr Psychiatry.

[56] Sacks O (2012). Hallucinations.

[57] Seidel K, Mahlke J, Siswanto S (2015). The brainstem pathologies of Parkinson’s disease and dementia with Lewy bodies. Brain Pathol.

[58] Shine JM, Halliday GM, Naismith SL (2011). Visual misperceptions and hallucinations in Parkinson’s disease: dysfunction of attentional control networks?. Mov Disord.

[59] Teunisse RJ, Zitman FG, Cruysberg JRM (1996). Visual hallucinations in psychologically normal people: Charles Bonnet’s syndrome. Lancet.

[60] van der Gaag M, Valmaggia LR, Smit F (2014). The effects of individually tailored formulation-based cognitive behavioral therapy in auditory hallucinations and delusions: a meta-analysis. Schizophr Res.

[61] Visser F, Vlaar AMM (2019). Diplopia in Parkinson’s disease: visual illusion or oculomotor impairment?. J Neurol.

[62] Walpola IC, Muller AJ, Hall JM (2020). Mind-wandering in Parkinson’s disease hallucinations reflects primary visual and default network coupling. Cortex.

[63] Weil RS, Schrag AE, Warren JD (2016). Visual dysfunction in Parkinson’s disease. Brain.

[64] Wood RA, Hopkins SA, Moodley KK (2015). Fifty percent prevalence of extracampine hallucinations in Parkinson’s disease patients. Front Neurol.

[65] Zarkali A, Lees AJ, Weil RS (2019). Flickering stimuli do not reliably induce visual hallucinations in Parkinson’s disease. J Parkinsons Dis.

